# *N*,*N*-Bis(9-methyl-3-carbazolyl)-4-anisidine as an Electroactive Material for Use in Perovskite Solar Cells

**DOI:** 10.1021/acsaem.3c00102

**Published:** 2023-05-16

**Authors:** Jonas Keruckas, Patryk Janasik, Rasa Keruckienė, Pawel Czulkin, Malgorzata Czichy, Mieczyslaw Lapkowski, Dmytro Volyniuk, Ranush Durgaryan, Byeong Jo Kim, Gerrit Boschloo, Juozas Vidas Gražulevičius

**Affiliations:** † Department of Polymer Chemistry and Technology, Kaunas University of Technology, Baršausko 59, Kaunas 51423, Lithuania; ‡ Department of Physical Chemistry and Technology of Polymers, Silesian University of Technology, Strzody 9, Gliwice 44-100, Poland; § Centre for Organic and Nanohybrid Electronics, Silesian University of Technology, Konarskiego 22b, 44-100 Gliwice, Poland; ∥ Centre of Polymer and Carbon Materials, Polish Academy of Sciences Zabrze, M. Curie-Sklodowskiej 34, 41-819 Zabrze, Poland; ⊥ Department of Chemistry - Ångström Laboratory, Physical Chemistry, Uppsala University, Ångströmlaboratoriet, Lägerhyddsvägen 1, 751 20 Uppsala, Sweden

**Keywords:** perovskite solar cell, time-of-flight, electrooxidation mechanism, EIS, DFT

## Abstract

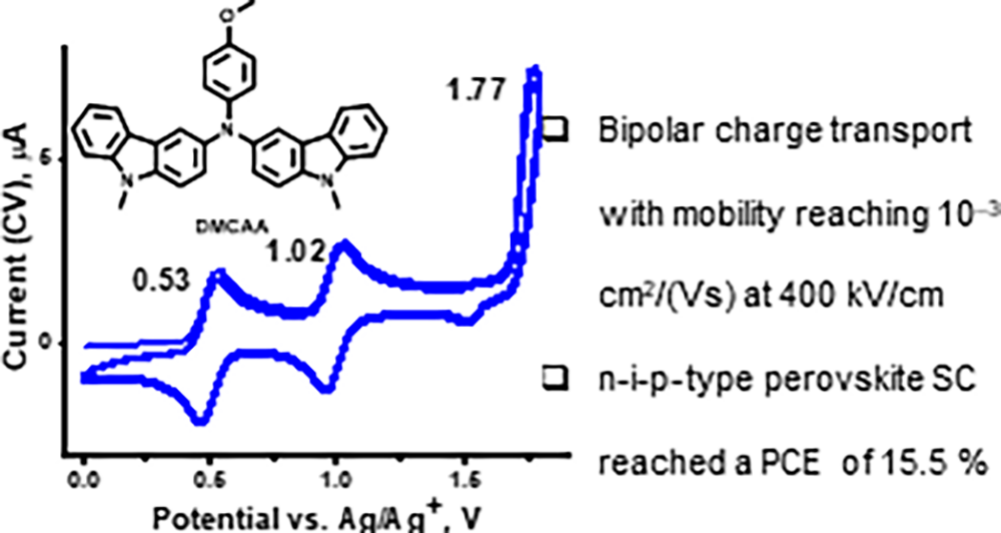

Di(9-methyl-3-carbazolyl)-(4-anisyl)amine is presented as an effective hole-transporting material suitable for application in perovskite solar cells. It is obtained by a three-step synthesis from inexpensive starting compounds. It has a relatively high glass transition temperature of 93 °C and thermal stability with 5% weight loss at 374 °C. The compound exhibits reversible double-wave electrochemical oxidation below +1.5 V and polymerization at higher potential. A mechanism for its oxidation is proposed based on electrochemical impedance and electron spin resonance spectroscopy investigations, ultraviolet–visible–near-infrared absorption spectroelectrochemistry results, and density functional theory-based calculations. Vacuum-deposited films of the compound are characterized by a low ionization potential of 5.02 ± 0.06 eV and hole mobility of 10^–3^ cm^2^/(Vs) at an electric field of 4 × 10^5^ V/cm. The newly synthesized compound has been used to fabricate dopant-free hole-transporting layers in perovskite solar cells. A power conversion efficiency of 15.5% was achieved in a preliminary study.

## 1 Introduction

Carbazole is a heteroaromatic moiety that is commonly used in the design of organic compounds utilized for electronic applications. For more than 20 years, *N*-linked carbazoles have appeared in the structures of popular hole-transporting and energy-transfer materials (referred to as “hosts”) used in organic light-emitting devices (OLEDs).^1−3^

The first reports on amino-substituted carbazoles appeared in the early 2000s.^4^ These were 3,6-bis(diphenylamino)carbazoles that are easily synthesized from inexpensive and commercially available starting materials. They are characterized by ionization potentials (IPs) of *ca.* 5.3 eV, hole mobilities of an order of about 10^–4^ cm^2^/(Vs), and glass-transition temperatures (*T*_g_) of *ca.* 90 or 110 °C depending, respectively, on either ethyl or phenyl substituents at the *N*-9-position of carbazole. Their di(*p*-tolyl)amino-substituted analogues have slightly lower IPs (5.15–5.3 eV) and higher potential of charge transport: the hole mobilities in 1:1 mixtures with bisphenol-Z polycarbonate reach 10^–4^ cm^2^/(Vs),^5^ implying that they can be higher by 1–2 orders of magnitude in the neat layers. Nearly a decade ago, the structure of 3,6-bis[di(4-anisyl)amino]carbazole was introduced as a very promising fragment of the compounds intended for photovoltaic applications.^6^ It has an IP as low as 5.0 eV and hole mobility reaching 3 × 10^–3^ cm^2^/(Vs). Later on, tens of modifications of this type of structure were developed for applications as hole transporting materials (HTMs) in both dye-sensitized solar cells^7,8^ and perovskite solar cells (PSCs), which afforded a power conversion efficiency (PCE) of the latter of more than 20%.^9−14^ The linking of two 3,6-bis[di(4-anisyl)amino]carbazole fragments through aromatic bridges, particularly those having electron donor character, has some positive effect since PCEs above 20% were achieved for these HTMs in PSCs.^13,15−17^ Considering the relatively complicated synthesis of such compounds and the improvement in absolute PCE only by 1–2%, such compounds and PSCs containing their layers are expected to be considerably less cost-effective. The properties of mono-substituted (diphenylamino)carbazoles were similar.^9^ Some of these materials have found application in OLEDs,^18^ and some of them have been tested in dye-sensitized solar cells.^9^ However, such types of structures have two obvious disadvantages. First, the preparation of asymmetric mono-substituted carbazoles is more complicated than that of symmetrical di-substituted counterparts, and lower yields of intermediates are obtained. Second, because of their lower molecular weight, their *T*_g_ values are lower than those of di-substituted analogues. The *T*_g_ values of the reported compounds vary in the range of 40–70 °C depending on the substitution at the *N*-position of carbazole (either alkyl or aryl) and the substituents attached to the diphenylamine unit. The *T*_g_ value is regarded as the criterion for the morphological stability of the active amorphous layers of the optoelectronic devices.^20^

Recently, several studies have been performed on tris(carbazolyl)amines that revealed that they are not only efficient charge-transporting and energy-transferring materials^21,22^ but also good emitters that exhibit delayed fluorescence with quantum yields of *ca.* 60–90%.^23^

To date, only a few reports on di(carbazolyl)anilines have been known.^24^ They are characterized by low ionization potentials of *ca.* 5.0 eV, hole mobilities of *ca.* 10^–4^ cm^2^/(Vs),^25^ and *T*_g_ values slightly above 100 °C. The use of longer alkyl chains significantly reduces their *T*_g_ (*e.g.*, *T*_g_ of 40 °C is observed for the compound bearing the *N*-(ethylhexyl)carbazole moiety^25^), while the remaining properties are only slightly affected. Compared with tris(carbazolyl)amines, the synthesis of di(carbazolyl)arylamines can be more cost-effective since anilines and some other commercially available aromatic amines (e.g., aminonaphthalenes) are either considerably cheaper or easier to prepare than aminocarbazoles. Furthermore, the properties of di(carbazolyl)arylamines are similar to those of bis(diphenylamino)carbazoles, which appear to be the most promising among all of the discussed.

In this study, we aimed to develop an easily synthesizable organic semiconductor for PSCs with a low-ionization potential and good hole-transporting properties.

## 2 Experimental Section

The detailed descriptions of equipment and the procedures of measurements, as well as the synthesis and isolation of the compounds together with their structural characterization, are given in the Supporting Information.

## 3 Results and Discussion

### 3.1 Synthesis and Characterization

**Figure 1:**
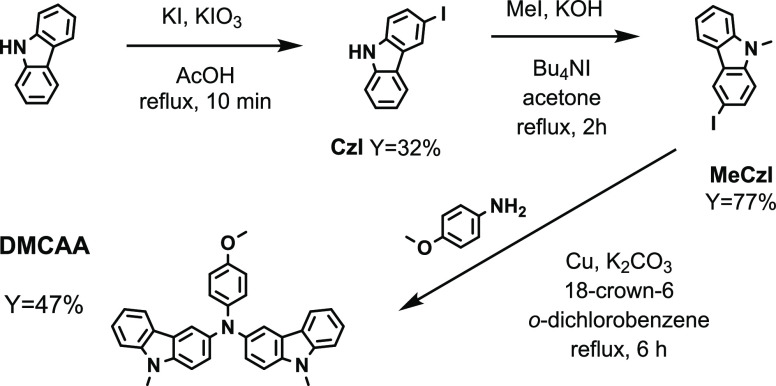
Synthetic route to *N*,*N*-bis(9-methyl-3-carbazolyl)-4-anisidine (**DMCAA**).

The synthetic route to *N*,*N*-bis(9-methyl-3-carbazolyl)-4-anisidine (**DMCAA**) is presented in Figure 1. The intermediate compounds, 3-iodo-9*H*-carbazole (**CzI**) and its 9-methyl-substituted derivative (**MeCzI**), were subsequently prepared from 9*H*-carbazole according to the procedures found in the literature.^26,27^**DMCAA** was obtained by the modified Ullmann condensation of **MeCzI** with *p*-anisidine according to the reported procedure.^28^

The intermediate compounds **CzI** and **MeCzI** were obtained in *ca.* 30 and 80% yields, respectively. The target compound (**DMCAA**) was obtained in *ca.* 50% yield. The structure of the compound was confirmed by NMR, IR spectroscopies, and mass spectrometry. The NMR samples of benzene-*d*_6_ and acetone-*d*_6_ solutions were investigated to confirm the stability of the target compound. The spectra of both the solutions were found to be identical. **DMCAA** dissolves easily in common organic solvents such as acetonitrile. Alcohols and saturated hydrocarbons do not dissolve it.

### 3.2 Thermal Properties

**Figure 2:**
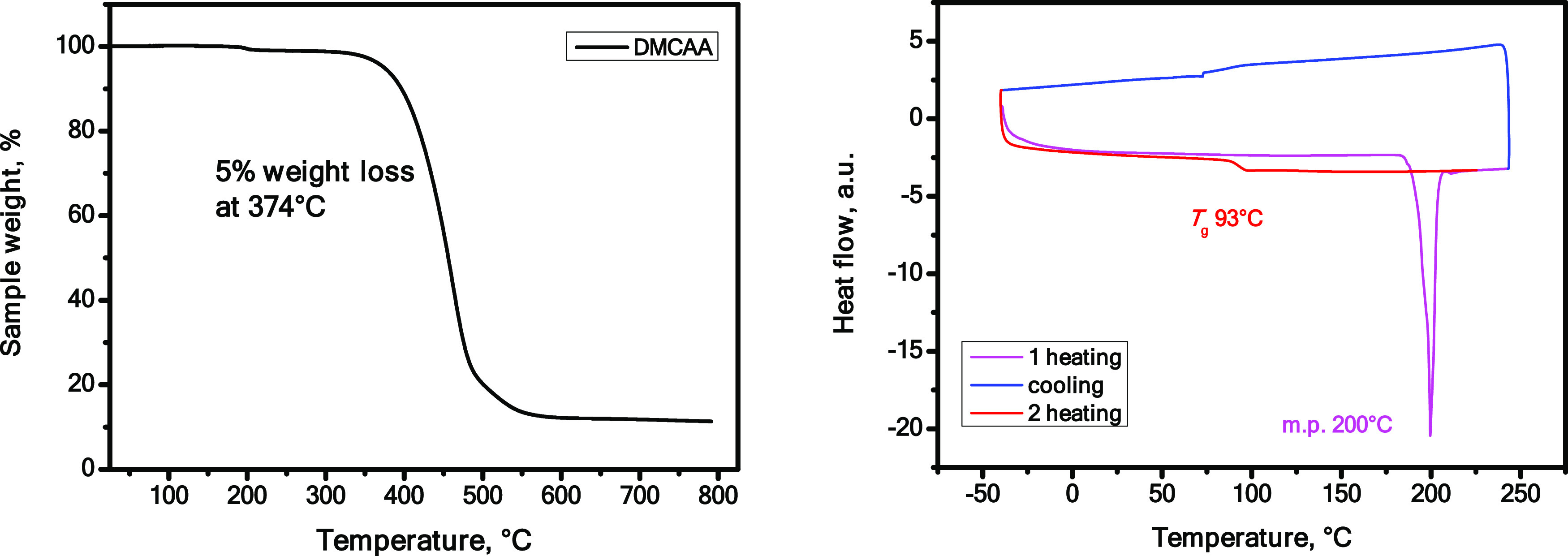
TGA (left) and DSC (right) curves of **DMCAA.**

The behavior of **DMCAA** under heating was investigated by thermogravimetric analysis (TGA) and differential scanning calorimetry (DSC). Figure 2 depicts the TGA and DSC curves. A sample of **DMCAA** begins to lose its weight above 300 °C. Its 5% weight loss is set at 374 °C. At the initial stage, the process appears to be sublimation because the decrease in weight occurs in the single stage. When the temperature approaches 500 °C, a curve bend at *ca.* 20% of the initial weight points to the thermal degradation of the compound. This hypothesis is supported by a charcoal remainder, which comprises more than 10% of the initial sample weight.

As evidenced by a sharp endothermic peak during the first DSC heating, a solid sample of **DMCAA** melts at 200 °C. On the second heating scan, a hump of the glass-to-liquid transition is visible at 93 °C, which is attributed as its glass transition temperature (*T*_g_). The investigation of thermal properties leads to the conclusion that *T*_g_ and thermal stability are adequate for practical applications. Furthermore, it can be both solution-casted and deposited by vacuum evaporation at temperatures below 350 °C.

### 3.3 Photoelectrical Properties

Ionization potential and charge-drift mobilities were determined for solid layers of **DMCAA** by photoelectron emission (PE) and time-of-flight (TOF) techniques, respectively.

**Figure 3:**
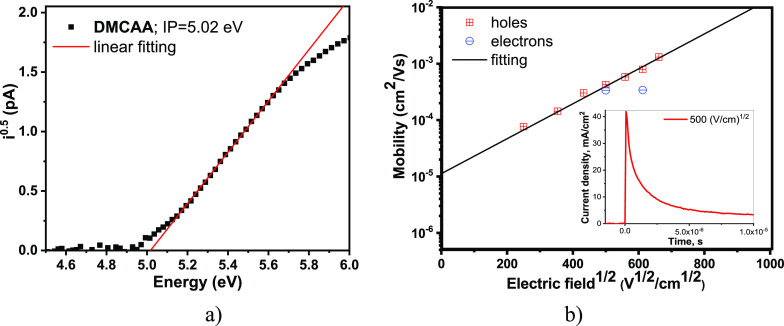
Photoelectron emission spectrum (a) and electric field dependence of charge mobility (b) for the solid layers of **DMCAA.** The inset shows the TOF current transient in linear scales for holes for the solid layer of **DMCAA** at an applied electric field of 40 V (square-root electric field of 500 (V/cm)^1/2^).

The photoelectron emission spectrum (Figure 3a) indicates that the solid-state IP^PE^ of **DMCAA** is as low as 5.02 ± 0.06 eV. To obtain this value, the linear part of the photoelectron emission spectrum was extrapolated to the baseline by linear fitting. The error of fitting was very low (*R*^2^ = 0.999).

The charge transport in the vacuum-deposited layer of **DMCAA** was found to be highly dispersive. Transient times of charge carriers could only be detected using the double logarithmic scale (Figures S10 and S11). As shown in Figure 3b, the hole mobility in **DMCAA** changes from *ca.* 10^–4^ cm^2^/(Vs) at 100 kV/cm to 10^–3^ cm^2^/(Vs) at 400 kV/cm. The obtained square-root field dependence of the mobility for holes is in very good agreement with the Poole–Frenkel prediction μ = μ_0_ exp β*E*^1/2^. The zero electric field mobility (μ_0_) was estimated from the interception of the fitting curves with the axis *Y* at zero electric field (*E* = 0), and the Poole–Frenkel electric field dependence (β) was obtained from the slopes of fitting curves using the relation slope = β. According to the Poole–Frenkel fitting, μ_0_ = 1.1 × 10^–5^ cm^2^/Vs and β = 8.86 × 10^–3^ (cm/V)^1/2^. The fitting error was low (*R*^2^ = 0.985), mainly representing the error in determination of the transit time at the different electric fields (Figure S10). Despite the strong electron-donating character of **DMCAA,** electron transport was also detected. Unfortunately, the transient times for electrons could be set only at two points with different electric fields (Figure S11) and the reliability of the data was rather low. However, it can be concluded from these data that electron mobility reaches about 3 × 10^–4^ cm^2^/(Vs) at electric field between 250 and 400 kV/cm and is close to that of holes.

### 3.4 Electrochemical and Spectroelectrochemical Investigations with Quantum Mechanical Modeling

**Figure 4:**
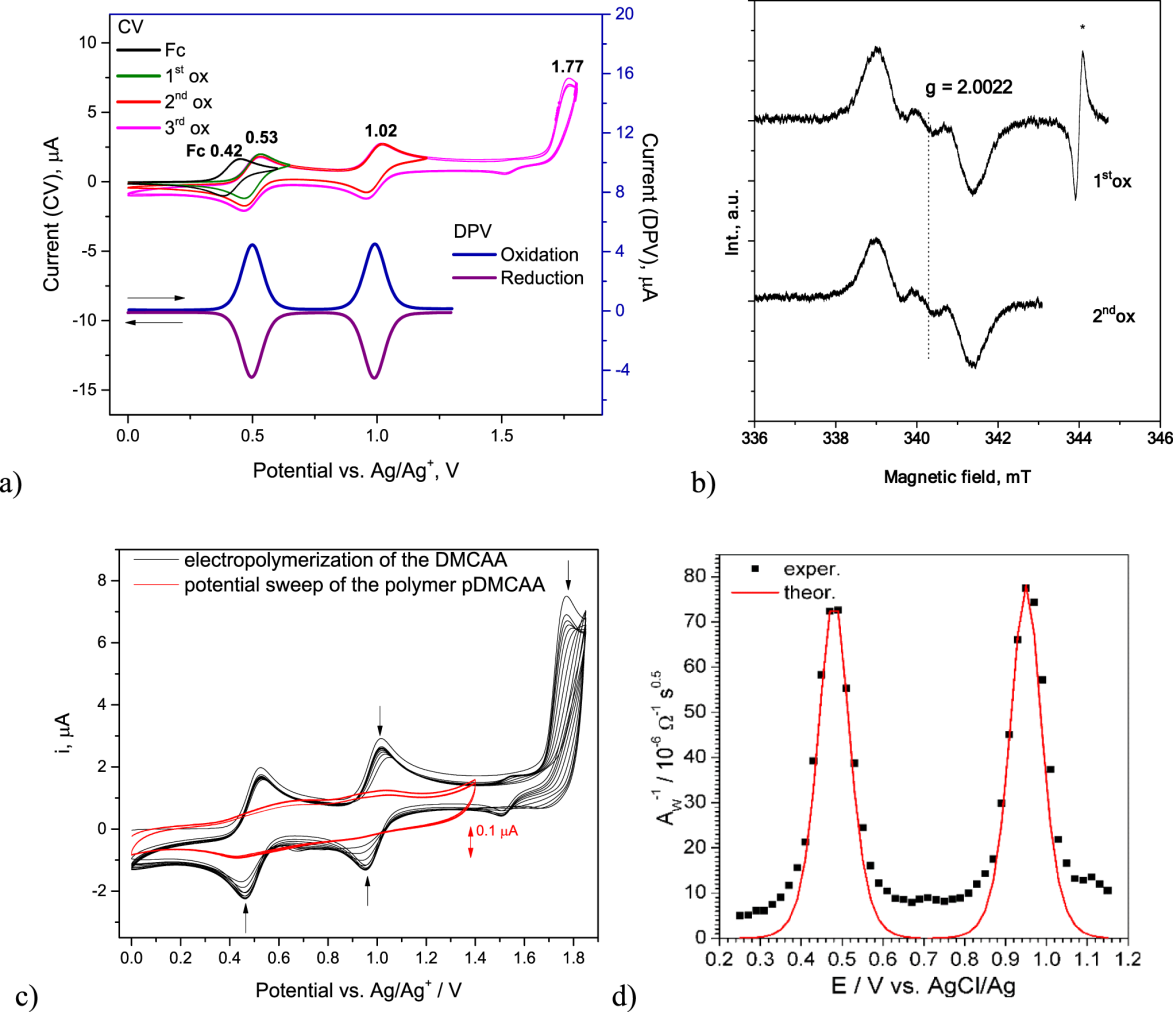
Cyclic voltammograms (CV) after the first, second, and third anodic peaks and differential pulse voltammograms (DPV) with the first and second anodic peaks (a); ESR spectra at the first and second anodic peaks (b) and CVs of electropolymerization after the third anodic peak recorded for 10^–3^ M solution of **DMCAA** in acetonitrile/tetrabutylammonium hexafluorophosphate TBAHFP (0.1 M). The scan rate for CVs was equal to 0.1 V/s (c). Dependence of the inverse Warburg constant and individual fitting of each peak with eq S6 (d).

It was established by cyclic voltammetry (CV) and differential pulse voltammograms (DPV) that **DMCAA** undergoes a three-step oxidation process (Figure 4a). The first two oxidation peaks are completely reversible with maximum potentials at 0.53 (**1st**) and 1.02 V (**2nd**) vs Ag|Ag^+^ (Figure 4a, CV). Electropolymerization was only found to occur above 1.77 V, i.e., after the third oxidation peak (**3rd**). The polymer **pDMCAA** also exhibits the first two reversible redox pairs (Figure 4c). The CV voltammogram of **DMCAA** electropolymerization is very similar to that of tris(9-ethyl-3-carbazolyl)amine, which we previously reported^29^ and indicates a coupling process by carbazole units. The low potential of the **1st** most likely indicates the first oxidation of the amino group.^30,31^ Also, carbazoles with appropriate amino substituents can achieve an oxidation potential of 0.38 V vs Ag/Ag^+^, which is lower than the first (**1st**) oxidation potential of **DMCAA**.^32^

Further, electrochemical impedance spectroscopy (EIS), electron spin resonance (ESR), and ultraviolet–visible–near-infrared absorption spectroelectrochemistry investigations, as well as geometry optimization using the density functional theory (DFT)/time-dependent DFT (TDDFT) method, were carried out to propose the mechanism of electropolymerization. The charge ratios under the current/potential curves can only be compared between reversible processes, and charges in process **1st** and **2nd** are equal. The obtained EIS spectra (Figure S3) were found to be consistent with a simplified Randles model of diffusion-controlled charge transfer.^33−35^ The dependence of the reverse Warburg constant versus potential (*A*_W_–*E*) was fitted with a theoretical function (eq S6) by adjusting the parameters of the process, that is, a standard redox potential *E*° of the first and second step, an average diffusion coefficient *D* of 1.35 × 10^–9^ m^2^/s, and the number of electrons transferred within each step (*z*) of 1 (Figure 4d). In the first step, one electron is taken, and in the following step, another one, a total of two electrons, and even then, the oxidized **DMCAA** molecule is not a reactive, which had to be explained further.

**Figure 5:**
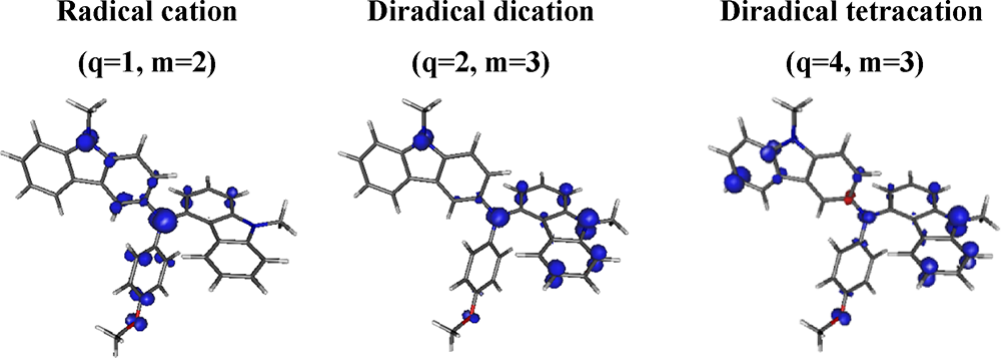
Spin density of oxidized forms of **DMCAA** calculated at B3LYP/6-31G(d)/CPCM(acetonitrile). The isovalue is equal to 0.005 e^–^/au^3^.

B3LYP 6-31G(d)/CPCM calculations confirmed the high lying HOMO level of **DMCAA** with an energy of −4.69 eV. This observation agrees with the results of electrochemical measurements because the potential for the onset of oxidation is 0.28 V, which corresponds to a HOMO energy of −4.82 eV, so close to this theoretical one. The energies of the subsequent HOMO levels are −5.48 eV (HOMO-1) and −5.67 eV (HOMO-2). The HOMO orbital is located on the phenyl unit of an amine group and on the benzene rings of both carbazoles from the amide side (Figure S11). Therefore, the amino group is definitely involved in the first stage of oxidation. The LUMO orbital is located in both carbazole moieties, and the energy of this orbital (−0.81 eV) is sufficiently high that no reduction of the monomer is observed during electrochemical analysis. The spin density of **DMCAA** of subsequent oxidation states was also determined (Figure 5). The spin density of the oxidized states can reveal information about coupling positions due to oxidation. The formation of a diradical dication following the radical–cation state indicates the location of the spin density on the central unit and one of the carbazole moieties. As a result, oxidation of one carbazole unit does not initiate polymerization. In turn, the highly oxidized state of the diradical tetracation increases the spin density at the key positions of carbazole, i.e., three and six of both terminal segments, which likely promotes the electropolymerization process.

**Figure 6:**
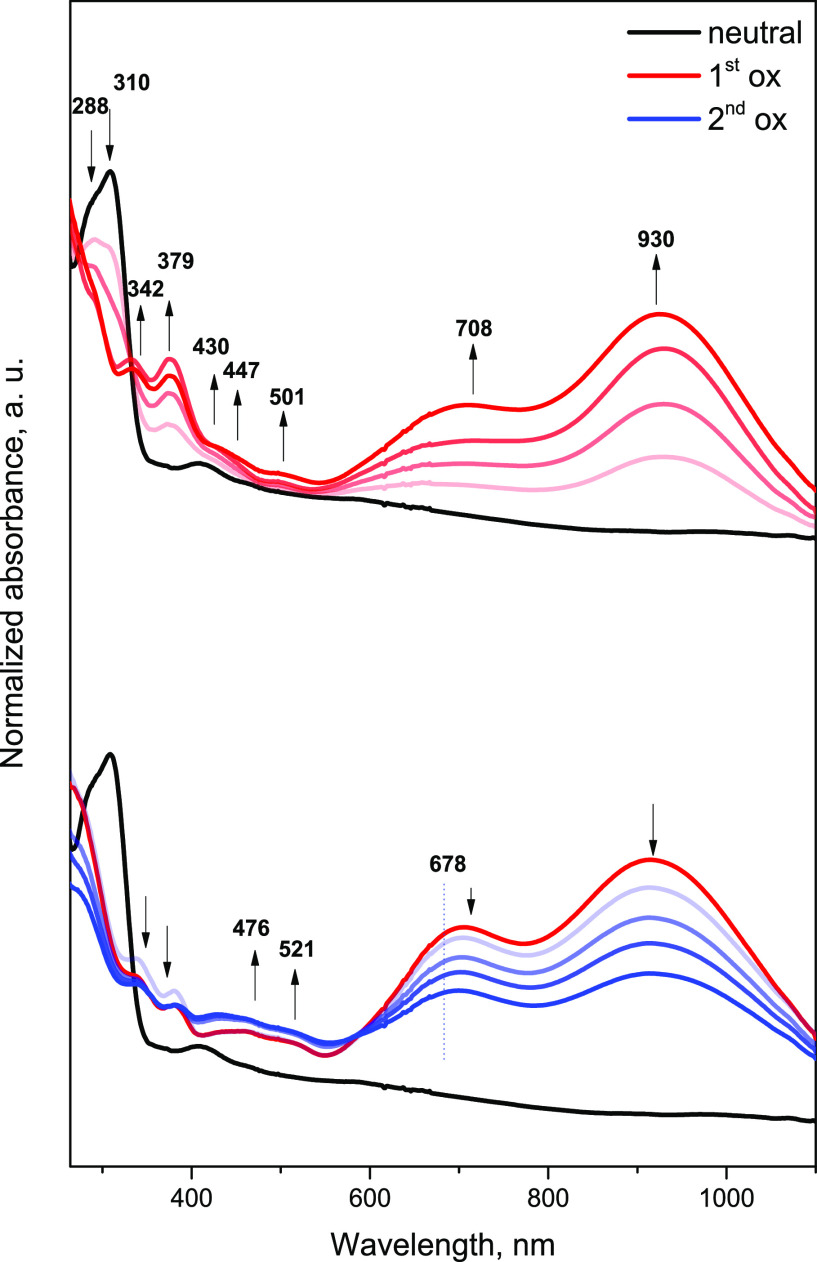
Results of UV–vis–NIR spectroelectrochemistry measurements for 10^–3^ M solution of **DMCAA** in acetonitrile/tetrabutylammonium hexafluorophosphate TBAHFP (0.1 M). The colors indicate the recorded spectrum at the specific potentials in the neutral state (neutral), the first oxidation state (1st ox), and the second oxidation state (2nd ox). The lighter colors correspond to the gradual achievement of the necessary potential to generate a given form of the compound.

The results of EIS and the calculations of the spin density of the oxidized forms (B3LYP/6-31G(d)/CPCM(MeCN)) show the formation of the radical cation and diradical dication at the first and second anodic peaks, respectively. UV–vis spectroelectrochemical measurements reveal the formation of bands at 708 and 930 nm in the region of the first oxidation peak (Figure 6, red curve).

**Figure 7:**
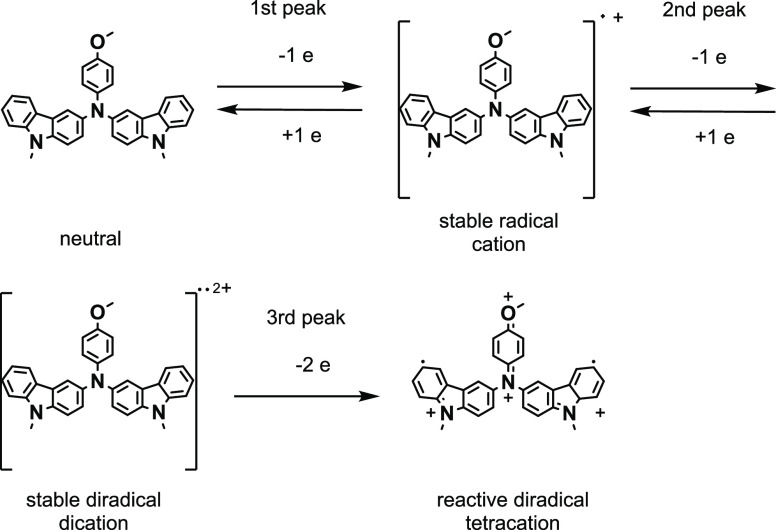
Proposed mechanism of electrooxidation of **DMCAA** during the subsequent first, second, and third anodic peak of cyclic voltammograms (CV).

After reaching the second oxidation peak potential, the 930 nm band decreases followed by a smaller decrease, while the 678 nm band is maintained. Additionally, there is a further increase in absorbance between 400 and 550 nm, which suggests the presence of a higher oxidation state (Figure 6, blue curve). Comparing the experimental UV–vis spectra with those generated by quantum chemical calculations (Figure S13), the similarities can be observed for the first oxidation stage (**1st**). The disappearance of the 310 nm band in the spectrum of the neutral form indicates a chemical change in the nitrogen atoms in the triaminic subunits.^34^ The additional bands observed at 708 and 930 nm in the experimental measurement confirm the formation of a radical cation in the first stage of oxidation, where in the computational spectrum, they occur at 727 and 905 nm, respectively. In the second stage of oxidation (**2nd**), the decrease in the intensity of the 930 nm band can be observed, which also corresponds to the computational spectrum, where this band disappears. The additional disappearance of bands in the experimental spectrum below 400 nm indicates the complete excitation of the molecule. This observation also corresponds to the computational spectrum for the diradical dication, where all bands below 400 nm disappear. The calculations shown in the previous part of the article also indicate that the entire molecule is excited. This is because the HOMO and electron spin density are distributed throughout the entire molecule, suggesting that the entire molecule is involved in the excitation process. The ESR technique made it possible to determine paramagnetic states, types of spin, and degree of spin delocalization of the **1st** and **2nd** states. The *g* factors for the ESR signals recorded at the potentials of the first and second oxidation peaks (Figure 4b) indicate high electron delocalization (*g* = 2.0022) for these moieties, which could explain their non-reactivity.^36^ The proposed mechanism of electrochemically performed oxidation is shown in Figure 7. Only the oxidation of **DMCAA** with the removal of four electrons causes the stronger localization of electrons on both terminal benzene rings in carbazole segments, increasing the efficiency of the addition processes between diradical tetracations of **DMCAA**. Although dications are non-reactive states, stable radical forms can also be indicated, which has just been verified.

### 3.5 Application in Perovskite Solar Cells

**Figure 8:**
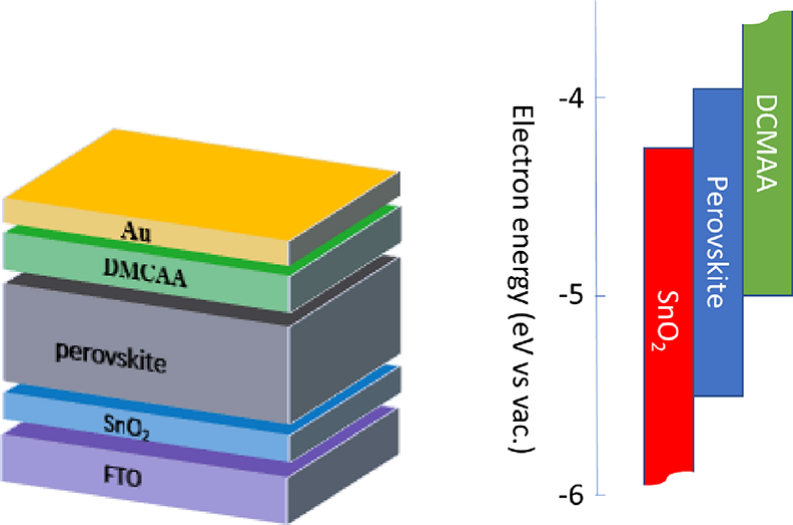
General configuration of the perovskite solar cell and energy level scheme of the device.

**DMCAA** was employed as a dopant-free HTM in PSCs with the n–i–p device structure, see Figure 8. A thin layer of **DMCAA** was deposited on top of the perovskite film by means of spin coating. A detailed description of the device fabrication is given in the Supporting Information.

PSCs consisting of the following layers were fabricated: a fluorine-doped tin oxide (FTO)-coated glass substrate, SnO_2_, perovskite, HTM, and a gold electrode. To compare the performance of **DMCAA** with that of conventional HTMs, also, devices with spiro-OMeTAD were prepared, both without dopants and with three different dopants,^37,38^ namely, 4-*tert*-butylpyridine (tBP), Li-bis(trifuoromethanesulphonyl)imide (Li-TFSI), and tris(2-(1*H*-pyrazol-1-yl)-4-*tert*-butylpyridine)cobalt(III) (FK-209). These dopants (molar ratio spiro-OMeTAD:TBP:Li-TFSI:FK209 of 1:3.3:0.5:0.05)^39^ are known to partially oxidize spiro-OMeTAD, thus not only increasing carrier density but also improving carrier mobility. On the downside, however, they are also detrimental to the device stability, which is the reason for investigating dopant-free HTMs.

**Figure 9:**
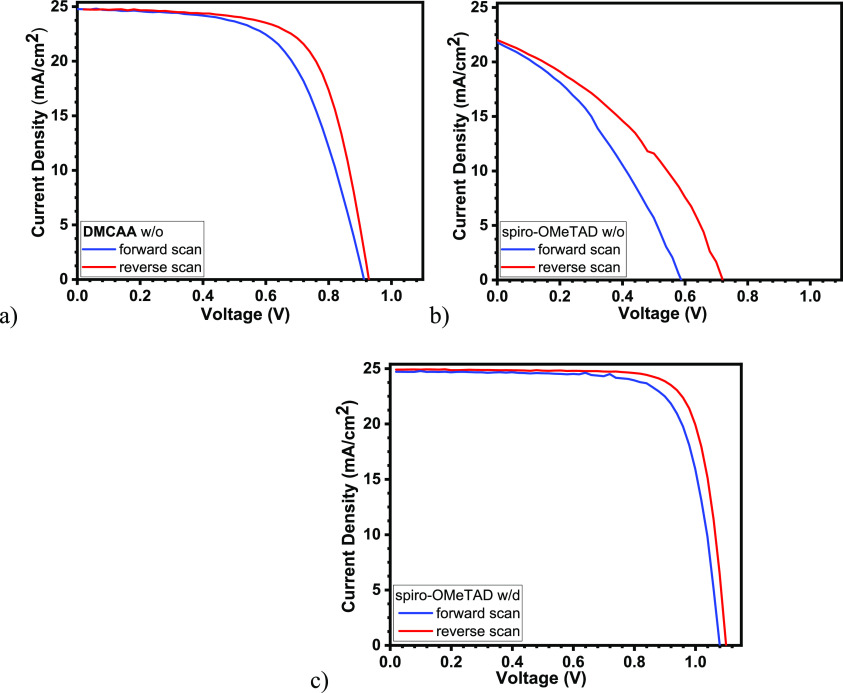
(a) *J*–*V* characteristics (forward and reverse measurements) of the PSC with additive-free **DMCAA** as HTM; (b) same with additive-free spiro-OMeTAD as HTM; (c) same with spiro-OMeTAD with additives. The composition of the perovskite is FA_0.91_MA_0.09_PbI_3_.

Table 1 summarizes the photovoltaic characteristics of the best performing PSC using **DMCAA** as an HTM. Figure 9 and Figure S13 show the corresponding current density–voltage (*J*–*V*) curves. PSCs with dopant-free HTMs based on **DMCAA** and spiro-OMeTAD exhibited PCEs of 15.53 and 4.98%, respectively. The **DMCAA**-based device exhibited a PCE that was 3.3 times higher than that of the spiro-OMeTAD-based PSC. Due to its high resistivity, the device with the layer of nondoped spiro-OMeTAD had a fill factor much lower than that of the device with the layer of nondoped **DMCAA**, which we attribute to the much higher resistivity of pristine spiro-OMeTAD compared to **DMCAA**. Interestingly, the open-circuit potentials of these devices differed greatly, where the **DMCAA** devices gave a higher *V*_OC_ by more than 200 mV. Nevertheless, HTMs with and without dopants do not have the same energy level, resulting in a lower *V*_OC_ in some cases. The PSC with the **DMCAA** layer without dopants showed a lower performance but was comparable with that of the device containing the doped spiro-OMeTAD layer. The control device with spiro-OMeTAD containing all additives showed a maximum PCE of 21.65% (see Figure 9c and Table 1). Notably, it had an open circuit significantly higher than dopant-free **DMCAA** and spiro-OMeTAD devices. The most likely explanation is the additional passivation of the interface due to the 4-*tert*-butylpyridine used as an additive in the doped-spiro-OMeTAD devices.

**Table 1:** Photovoltaic Parameters Determined from *J*–*V* Measurements (Forward Scan) of PSCs Containing **DMCAA** and spiro-OMeTAD as HTMs^a^

HTMs	PCE (%)	FF (%)	*J*_SC_ (mA/cm^2^)	*V*_OC_ (V)
**DMCAA**	15.53	67.59	24.75	0.93
spiro-OMeTAD w/o^b^	4.98	37.54	21.46	0.72
spiro-OMeTAD^c^	21.65	79.04	24.90	1.10

a For the best performing devices, obtained under a simulated AM 1.5G solar illumination of 100 mW cm^–2^. No anti-reflective coating was applied.

b spiro-OMeTAD prepared without additives.

c spiro-OMeTAD prepared with additives.

Furthermore, a few p–i–n-type devices were fabricated using dopant-free **DMCAA**. A PCE of 7.46%, along with an open-circuit voltage (*V*_OC_) of 0.72 V, a short-current density (*J*_SC_) of 22.63 mA/cm^2^, and fill factor (FF) of 53.21% was obtained. The structure of the device was as follows: glass/ITO/**DMCAA**/perovskite/PEABr/PCBM/BCP/Au. Although the performance was not very high, it demonstrates that **DMCAA** also has potential in p–i–n perovskite devices.

## 4 Conclusions

*N*,*N*-Bis(9-methyl-3-carbazolyl)-4-anisidine is reported as an efficient hole-transporting compound. The compound has thermal, electrochemical, and photoelectrical properties that make it suitable for an application in perovskite solar cells. Spectroelectrochemistry and electrochemical impedance spectroscopy studies performed together with the theoretical calculations suggested the compound electrooxidation via stable di- and tetracation intermediates formed in two reversible two-electron transfer processes. The electrochemical studies have provided insights on the influence of the charge distribution in the molecule over the overall characteristics of the hole-transporting compound. The demonstrated methodology gathering physical, chemical, and theoretical techniques can be applied to the further molecular design and advanced study of organic hole-transporting materials. The n–i–p-type perovskite solar cells with dopant-free HTM based on the introduced carbazole compound showed an encouraging light-to-electric power conversion efficiency of 15.5%.

## Supporting Information Available

Equivalent electric circuit (Figure S1); dependence of the inverse Warburg constant and individual fitting of each peak with eq S6 (Figure S2); ^1^H NMR spectrum of **DMCAA** in acetone-*d*_6_ (Figure S3); ^13^C NMR spectrum of **DMCAA** in acetone-*d*_6_ (Figure S4); ^1^H NMR spectrum of **DMCAA** in benzene-*d*_6_ (Figure S5); ^13^C NMR spectrum of **DMCAA** in benzene-*d*_6_ (Figure S6); mass spectrum (MS) of **DMCAA** (Figure S7); FT-IR spectrum of **DMCAA** (Figure S8); an establishment of transient times (ttr) for holes (positive current) and electrons (negative current) in the double logarithmic scale (both for time and current) from the kink of transient currents (Figures S9 and S10); shape of frontier orbitals and UV spectra of **DMCAA** calculated at B3LYP/6-31G(d)/CPCM (acetonitrile) (Figures S11 and S12); *J*–*V* characteristics of p–i–n-type devices **DMCAA** without additives (Figure S13) (PDF)
