# Supplementary material for: N,N-Bis(9-methyl-3-carbazolyl)-4-anisidine as an Electroactive Material for Use in Perovskite Solar Cells
Source: ACS Appl Energy Mater. 2023 May 16;6(11):5720–8. doi: 10.1021/acsaem.3c00102 (PMC10265720; doi:10.1021/acsaem.3c00102)
Supplement: Supplementary file 1 — ae3c00102_si_001.pdf [file ae3c00102_si_001.pdf]

Electronic Supplementary Information for:

***N*,*N*-Bis(9-methyl-3-carbazolyl)-4-anisidine as electroactive material for perovskite solar cells**

Jonas Keruckas[1], Patryk Janasik[2,3], Rasa Keruckienė[1,2], Pawel Czulkin[2,3], Malgorzata Czichy[2,3], Mieczyslaw Lapkowski[2,3,4]*, Dmytro Volyniuk[1], Ranush Durgaryan[1,5], Byeong Jo Kim[5], Gerrit Boschloo[5]*, Juozas Vidas Gražulevičius[1]*

[1]Department of Polymer Chemistry and Technology, Kaunas University of Technology, Baršausko 59, Kaunas, 51423, Lithuania

[2]Department of Physical Chemistry and Technology of Polymers, Silesian University of Technology, Strzody 9, Gliwice, 44-100, Poland

[3]Centre for Organic and Nanohybrid Electronics, Silesian University of Technology, Konarskiego 22b, 44-100 Gliwice, Poland

[4]Centre of Polymer and Carbon Materials, Polish Academy of Sciences Zabrze, M. Curie-Sklodowskiej 34, 41-819 Zabrze, Poland

[5]Department of Chemistry - Ångström Laboratory, Physical Chemistry, Uppsala University, Ångströmlaboratoriet, Lägerhyddsvägen 1, 751 20 Uppsala, Sweden

* Corresponding authors: Mieczyslaw.Lapkowski@polsl.pl, Juozas.Grazulevicius@ktu.lt, Gerrit.Boschloo@kemi.uu.se

Contents

Methods......2

Materials......7

Methods

DFT/TDDFT calculations were carried out with B3LYP [1] hybrid functional combined with 6-31G(d) basis set. Frequency calculations were systematically achieved (at the same level of theory) to confirm the minimum nature of the optimized geometries. All the calculations were performed using the ORCA 4.1.1. package programs [2]. Input files and molecular orbital plots were prepared with Gabedit 2.4.7 software [3]. The Conductor-like Polarizable Continuum Model (CPCM) was used to take into account the effect of solvent on the calculated structures [4].

Nuclear magnetic resonance (NMR) spectra were recorded on a Bruker Avance III spectrometer (400 MHz for ^{1}H and 100 MHz for ^{13}C). Mass spectra (MS) were obtained on a Waters ZQ 2000 spectrometer. Infra-red (IR) spectra of KBr pellets were recorded on a Vertex 70 Bruker spectrometer. Melting points were measured on a Krüss M3000 melting point meter.

Differential scanning calorimetry (DSC) measurements were performed on a TA Instruments DSC Q2000 equipment at heating/cooling rate of 10 °C/min under N_2 flow using an empty aluminium pan as a standard. Thermogravimetric analysis (TGA) was carried out using a TA Instruments TGA Q50 apparatus under N_2 flow at heating rate of 10 °C/min.

Solid-state ionization potential (IP) was established by photoelectron emission (PE) in air technique. A sample was prepared by vacuum-deposition of **DMCAA** layer on fluorine-doped tin oxide (FTO)-coated glass substrate. The sample was illuminated with a monochromatic radiation using an ASBN-D130-CM deep UV deuterium light source with a CM110 1/8m monochromator. A photocurrent occurred due to illumination was measured using a Keithley 6517B electrometer. The IP was estimated as the onset of photocurrent at a particular excitation energy value.

Charge-transporting properties were investigated using time-of-flight (TOF) technique [5]. A sample for TOF measurements was prepared by thermal deposition of **DMCAA** layer of 1.6 μm thickness on an indium-tin oxide (ITO) substrate under vacuum of 2×10^{-6} mBar and subsequently covering with aluminium layer (70 nm) under the same conditions. A pulsed Nd:YAG laser EKSPLA NL300 (wavelength 355 nm, pulse duration 3–6 ns) was used to generate charge carriers at ITO/organic surface by illumination through the ITO anode. A voltage at the sample surface was adjusted using a Keithley 6517B electrometer and the photocurrent transients were recorded on a Tektronix TDS 3032C oscilloscope. The carrier transit times (t_{tr}) were defined from TOF photocurrent transients plotted in double logarithmic scale. The carrier-drift mobility μ was calculated according to the equation $\mu = d^2/(U\times t_{tr})$, where d is the layer thickness, and U is the surface potential at the moment of illumination [6].

Cyclic voltammetry (CV) measurements were performed on a Bio-Logic SP-150 and a CH Instruments Electrochemical Analyzer model 620. A single-cell and three-electrode setup was used: 2 mm^2 platinum (Pt) disk as a working electrode, Pt coil as a counter electrode, and silver wire (Ag/Ag^+) as a pseudo-reference electrode calibrated with ferrocene as an external standard. The concentration of compound was 1.0 mM in acetonitrile in the presence of 0.1 M tetra-*n*-butylammonium hexafluorophosphate (TBAHFP) as a supporting electrolyte. Polymer films on a platinum electrode were prepared by electrooxidation. The potential was changed at a rate of 50 mV/s. Argon was bubbled before both reduction and oxidation.

The CV technique has also been used to measure the amount of electrons involved in an electrochemical reaction. 5 µL of a 0.5 M JK106 solution was dropped onto the ITO electrode and evaporated. Then, the CV measurement was carried out in a classic three-electrode cell ITO as working elekctrode, Pt coil as a counter electrode, and silver wire (Ag/Ag^+) as a pseudo-reference electrode calibrated with ferrocene as an external standard. Electrochemistry was carried out in water with the presence of 0.2 M sodium perchlorate as a supporting electrolyte. The potential was changed at a rate of 5 mV/s. Argon was bubbled before both reduction and oxidation. The formula used for the calculation is as follows:

$$z_{CV} = \frac{I_i C}{N_A V c^*} \quad \text{(S1)}$$

I_i - integrated current minus current for pure electrolyte in the range of 0.3 to 1.0 V vs. Ag/Ag^+ [A]

C - 6.24×10^{18} e

N_A – 6.02×10^{23} mol^{-1}

V – volume of dropped solution of **JK106** on ITO electrode [L].

c^* - concentration of solution of **JK106** dropped on ITO electrode [$molL^{-1}$].

the calculated value from the measurements came out to be 1.79 electrons (± 0.08), integrating the voltammetric curve in the range of two oxidation peaks. This gives approximately one electron per oxidation peak (z = 0,89 ≈ 1).

Differential pulse voltammetry (PDV) measurement was performed on a CH Instruments Electrochemical Analyzer model 620 using 2 mm^2 platinum (Pt) disk as a working electrode, Pt coil as a counter electrode, and silver wire (Ag/Ag^+) with modulation amplitude of 50 mV, modulation time of 30 ms, interval time of 200 ms, and step potential of 10 mV.

UV-Vis spectroelectrochemical measurements. Spectral changes accompanying electrooxidation were monitored *in situ* by thin-layer UV-Vis-NIR spectroscopy using an Ocean Optics QE6500 and a

NIRQuest apparatus equipped with DH-2000-BAL halogen and deuterium sources and detectors connected to an Autolab PGSTAT100N potentiostat. A thin optical cell with an optical path length of 0.08 mm was a quartz cuvette containing ITO/quartz electrode, PTFE separator (0.08 mm), silver wire (pseudo-reference electrode), and platinum mesh (counter electrode).

Electron spin resonance (ESR) spectra were acquired using a JEOL JES FA-200 X-band spectrometer with the following parameters: modulation width of 0.6 mT, microwave power of 1 mW, and amplitude of 300. A capillary quartz spectroelectrochemical cell was used equipped with a Pt wire working electrode in acetonitrile solution of the monomer (1 mM), Ag wire pseudo-reference electrode (calibrated versus E_o of Fc/Fc^+), and Pt coil counter electrode.

Electrochemical impedance spectroscopy (EIS) experiments were carried out using BioLogic SP150 potentiostat in 3 ml electrochemical cell with a Pt disc as a working electrode (diameter 1 mm), a Pt wire as a counter electrode, and a Ag wire as a reference electrode. The counter electrode was flame annealed before the experiment. The working electrode was cleaned by polishing. The reference electrode was preliminarily anodised in concentrated (3 M) KOH solution to cover its surface with silver oxide in order its potential to be stable in all experiments. All the potentials in this report are presented versus AgCl/Ag reference potential. A working acetonitrile solution containing 1 mM of organic compound and 0.1 M of tetra-*n*-butylammonium hexafluoroborate as a supporting electrolyte was used for all the experiments. The solution was saturated with argon for 20 min and the argon atmosphere was maintained during the whole experiment. Impedance spectra were obtained in 10kHz−1Hz frequency range with 20 points per decade in logarithmic scale (total number of frequencies in one spectrum was 61), AC voltage amplitude was 25 mV. The spectra were recorded in a potentiostatic staircase mode with 0.01 mV potential increment within 250 mV potential range that covered redox potential of the compound oxidation previously estimated from CV. The analysis of electrochemical impedance spectra and determination of equivalent circuit parameters was realized using EIS analyser program (http://www.abc.chemistry.bsu.by/vi/analyser).

Estimation of the number of redox-transferred electrons by EIS.

The obtained impedance spectra were found to be in accordance with a Randles model of diffusion-controlled charge transfer. The equivalent electric circuit is shown in **Figure 1**.

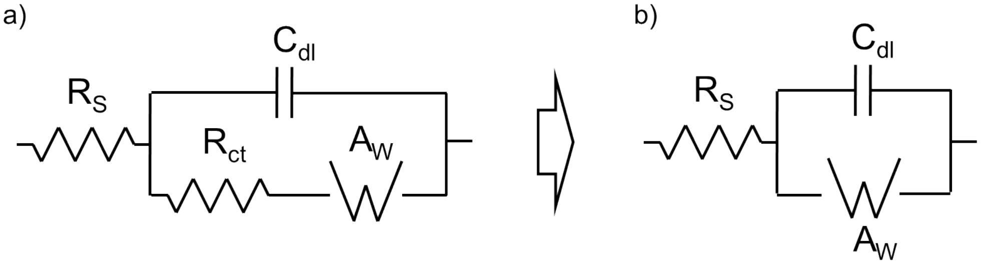

Figure S1. a: Equivalent circuit that was found to describe the studied redox process: R_s – series resistance, R_{ct} – charge transfer resistance, C_{dl} double layer capacitance, Z_W – diffusion impedance (Warburg element).
b: When charge transfer resistance is too small compared to Warburg constant (in the case of diffusion limitation) a simpler circuit can be used.

Besides the impossibility of charge transfer resistance estimation, we have used the Warburg coefficient to define the number of electrons transferred within the redox process.

The Warburg constant characterizes diffusion impedance

$$A_W = \frac{RT}{z^2F^2S\sqrt{2}}\left(\frac{1}{c_O^s\sqrt{D_O}} + \frac{1}{c_R^s\sqrt{D_R}}\right) \tag{S1}$$

where S is the electrode surface area, D_O, D_R are diffusion coefficients of oxidized and reduced forms, c_O^s, c_R^s are surface concentrations of electroactive species, which can be calculated according to the following formula:

$$c_O^s = \theta\frac{\xi c_O^b + c_R^b}{1 + \xi\theta} \tag{S2}$$

$$c_R^s = \frac{\xi c_O^b + c_R^b}{1 + \xi\theta} \tag{S3}$$

where c_O^b, c_R^b are bulk concentrations and

$$\xi = \sqrt{\frac{D_O}{D_R}} \tag{S4}$$

$$\theta = e^{\frac{zF}{RT}(E - E^\circ)} \tag{S5}$$

One may also assume that diffusion coefficients of both forms are equal. Of course, such assumption is not correct when the diffusion coefficient is the sought parameter, but in our case it is justified. Then combining the equations above and assuming equality of the diffusion coefficients one gets:

$$A_W^{-1} = \frac{z^2F^2c_\Sigma S\sqrt{2D}}{RT}\frac{\theta}{(1+\theta)^2} \quad \text{(S6)}$$

The number of transferred electrons **z** appears in the denominator of the equation and also defines the value of θ.

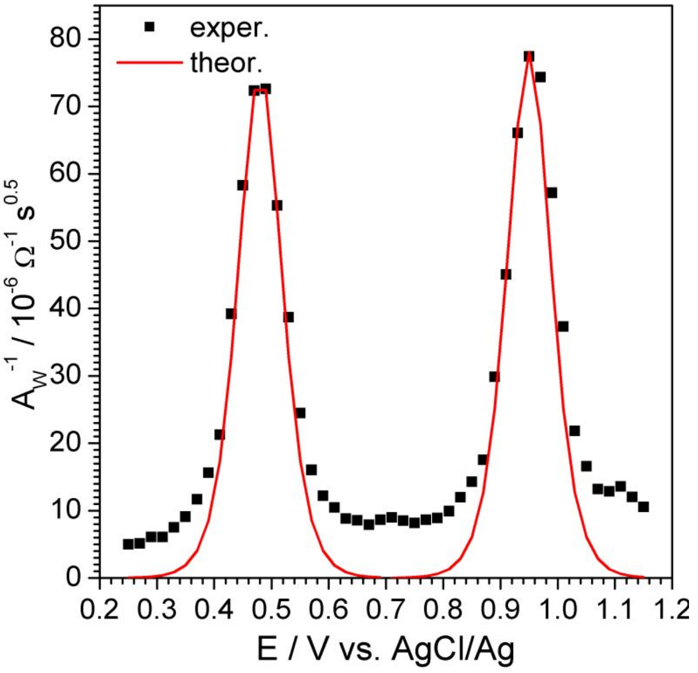

Figure S2. Dependence of the inverse Warburg constant and individual fitting of each peak with equation S6.

The values of parameters obtained from the fitting are as follows. For the first peak E^0 = 0.48 V, z = 1, D = $1.3 \cdot 10^{-9}$ $m^2 \cdot s^{-1}$. For the second peak E^0 = 0.95 V, z = 1, D = $1.4 \cdot 10^{-9}$ $m^2 \cdot s^{-1}$.

Perovskite solar cells fabrication. Planar heterojunction mixed perovskite with SnO_2 layer as ETL and DMCAA as HTM were fabricated by solution method. For the n-i-p architecture - the devices structure was: glass/FTO/SnO_2/$(FA0_{.91}MA_{0.09})PbI_3$/2D layer/ DMCAA /Au. FTO glass substrates were etched with zinc powder and hydrochloric acid (HCl 2 M) solution and washed with Hellmanex solution, acetone, ethanol and deionized water in an ultrasonic bath 30 min. Then, the substrates were cleaned by UV/ozone for 30min. ETL was deposited onto the substrates by spin coating a diluted solution of SnO_2 (the volume ration of water to SnO_2 colloid (Alfa-Aesar) was of 4:1) at 3000 rpm for 30 s in ambient air, then the substrates were annealed at 150 °C for 30 min. after cooling down, oxygen plasma cleaning for 25 minutes, the substrates were transferred into a nitrogen-filled glove box. The perovskite film was deposited by one spin-coating procedure., The solvent prepared deposited on the

top of substrate as followed lead (II) iodide (693 mg) was dissolved in the mixture of DMF and DMSO (9:1 volume ratio) and stirred overnight. Then the solution was spin coated on SnO_2 substrates at 1500 rpm for 20 s and annealed in the glovebox at 70 °C for 1 min.

Then mixed perovskite precursor solution, which was prepared by mixing of 80 mg formamidinium iodide (FAI), 8 mg of methylammonium iodide (MAI) and 5.7 mg of methylammonium chloride (MACl) in 1 mL of isopropyl alcohol and spin coated speed of 2000 rpm for 30 s and annealed at 140°C for 25 min in air. The resulting perovskite layer thickness is 600 – 700 nm. After cooling to room temperature a very thin layer of 2D perovskite was formed by spin coating phenethylammonium bromide (PEABr, 5 mg) dissolved in isopropyl alcohol (4 ml) (5000 rpm for 30 s) on top of the perovskite films, followed by brief heating. After cooling to room temperature, the prepared hole-conductor solution (0.02 mmol in 0.5469 ml CB) was subsequently deposited on top of the perovskite layer by spin coating at 4000 rpm for 30 s. Finally, an 80 nm gold film was deposited as back contact by thermal evaporation using a shadow mask to pattern the electrodes.

Materials

9*H*-Carbazole was inherited from *Reakhim* (former USSR), chloroform was obtained from *Chempur* (Poland), tetra-*n*-butylammonium iodide was inherited from *Reanal* (Hungary). Acetic acid, acetone, heptane, isopropanol, potassium carbonate, potassium hydroxide, potassium iodate, potassium iodide, and toluene were obtained from *Reachem* (Slovakia). 4-Anisidine, copper powder, 18-crown-6, *o*-dichlorobenzene, iodomethane, molecular sieves (3 Å), and silica-gel 60 were purchased either from Sigma-Aldrich, Alfa Aesar, or Fluka. The materials were used as received. Acetonitrile and dichloromethane used for electrochemical measurements were dried under molecular sieves and kept under inert atmosphere.

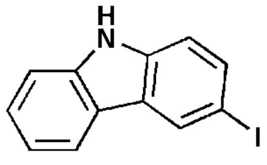

CzI

3-Iodo-9*H*-carbazole (**CzI**) was obtained by iodination of 0.1 mol (16.7 g) of 9*H*-carbazole as reported elsewhere [7]. Recrystallization from isopropanol afforded 9.4 g (32% yield) of small beige plates, mp 193–196°C (lit. 192–194°C [7]).

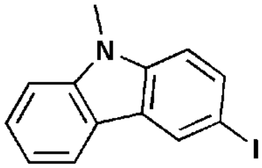

MeCzI

3-Iodo-9-methylcarbazole (**MeCzI**) was obtained by alkylation of 3-iodo-9*H*-carbazole by a similar procedure reported in [8]: 9.4 g (32 mmol) of **CzI** was dissolved in 100 ml of dry acetone, about 0.1 g of tetra-*n*-butylammonium iodide and an excess of iodomethane were added. The solution was heated to *ca.* 50°C and an excess of potassium hydroxide was added. After the complete consumption of **CzI** in about 2 hours (monitored by TLC), the mixture was filtered off and the solvent was distilled off by rotary evaporator under reduced pressure. The obtained brownish viscous oil was submitted to silica-gel 60 column and eluted with hexane. The obtained colourless oil was dried under vacuum and diluted with diethyl ether from which it has crystallized giving 7.6 g (yield 77%) of colourless small needles, mp 77–79°C (lit. 77–79°C [9]). ^{1}HNMR (acetone-d_6, 400 MHz) δ ppm: 3.75 (s, 3H, CH_3), 7.10 (t, 1H, J = 7.3 Hz), 7.26 (d, 1H, J = 8.6 Hz), 7.33–7.43 (m, 2H), 7.59 (d, 1H, J = 8.6 Hz), 8.03 (d, 1H, J = 7.8 Hz), 8.35 (d, 1H, J = 7.8 Hz).

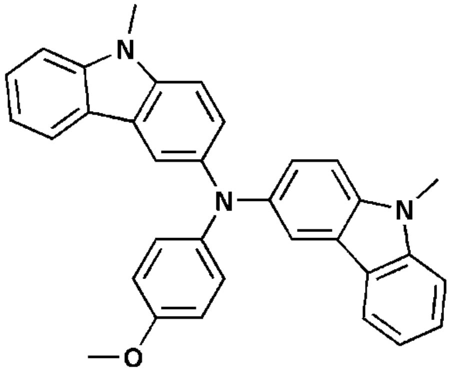

DMCAA ($C_{33}H_{27}N_3O$) M_r = 481.6

N,*N*-Bis(9-methyl-3-carbazolyl)-4-anisidine (**DMCAA**) was obtained by modified Ullmann condensation as described in the literature [10]: 1 g (8 mmol) of 4-anisidine, 7.4 g (24 mmol) of **MeCzI**, and 0.2 g of 18-crown-6 were dissolved in 50 ml of 1,2-dichlorobenzene. The mixture was heated above 100 °C under nitrogen and 4 g of potassium carbonate was added. When the temperature raised to 180 °C, 2 g of copper bronze were added and the reflux was continued for 6 hours. The reaction mixture was then cooled down and filtered through celite layer washing with chloroform. The solvents were removed by vacuum distillation. The dark solid resin was submitted to a silica-gel 60 column (5×15 cm) and eluted with heptane/toluene mixture 1:1 (v/v). A greenish-yellow resin obtained was recrystallized from isopropanol/chloroform mixture to give 1.82 g of yellow powder (yield 47%), m.p. 201–203 °C. ^{1}H NMR (acetone-d_6, 400 MHz) δ ppm: *1.91 (p, acetone)*, 3.63 (s, 3H, OCH_3), 3.75 (s,

6H, NCH_3), 6.70 (d, 2H, J = 9.0 Hz), 6.87 (d, 2H, J = 8.9 Hz), 6.96 (t, 2H, J = 7.4 Hz), 7.13 (dd, 2H, J = 8.7, 1.8 Hz), 7.28 (dd, 2H, J = 11.7, 4.3 Hz), 7.34 (dd, 4H, J = 15.4, 8.5 Hz), 7.72 (s, 2H), 7.82 (d, 2H, J = 7.8 Hz). ^{13}C NMR (acetone-d_6, 100 MHz) δ ppm: 28.46, *28.95 (sept, CD_3 acetone-d_6)*, 54.80, 108.78, 109.50, 114.43, 115.97, 118.50, 120.19, 122.44, 123.41, 123.57, 123.89, 125.67, 137.46, 141.63, 141.74, 143.54, 154.55, *205.26 (s, CO acetone-d_6)*. ^{1}H NMR (benzene-d_6, 400 MHz) δ ppm: 3.03 (s, 6H, NCH_3), 3.34 (s, 3H, OCH_3), 6.83 (d, 2H, J = 8.9 Hz), 7.00 (dd, 4H, J = 10.7, 8.6 Hz), 7.06 (t, 2H, J = 7.5 Hz), *7.15 (s, benzene)*, 7.34 (t, 4H, J = 7.4 Hz), 7.57 (d, 2H, J = 8.5 Hz), 7.68 (d, 2H, J = 7.8 Hz), 8.20 (s, 2H). ^{13}C NMR (benzene-d_6, 100 MHz) δ ppm: 28.10, 54.77, 108.31, 109.18, 114.82, 116.41, 118.70, 120.67, 122.90, 123.90, 124.00, 124.32, 125.53, *127.70 (t, benzene-d_6)*, 137.42, 141.58, 142.23, 143.91, 154.94. MS (ESI) *m/z*: 481.4 (*FW* calcd. for $C_{33}H_{27}N_3O$ = 481.6). FT-IR (KBr) *ν* cm^{-1}: 3048, 3028, 2993, 2929, 2906, 2879, 2831 (ν C–H), 1628, 1603, 1571, 1505, 1485, 1469, 1424 (ν C–C), 1360, 1312, 1291, 1273 (ν C–N), 1238 (ν C–O), 1178, 1149, 1121 ("fingerprints"), 1058, 1031 (ν C–O), 949, 927, 871, 853, 833, 805, 768, 744, 724, 634, 620 (γ C–H).

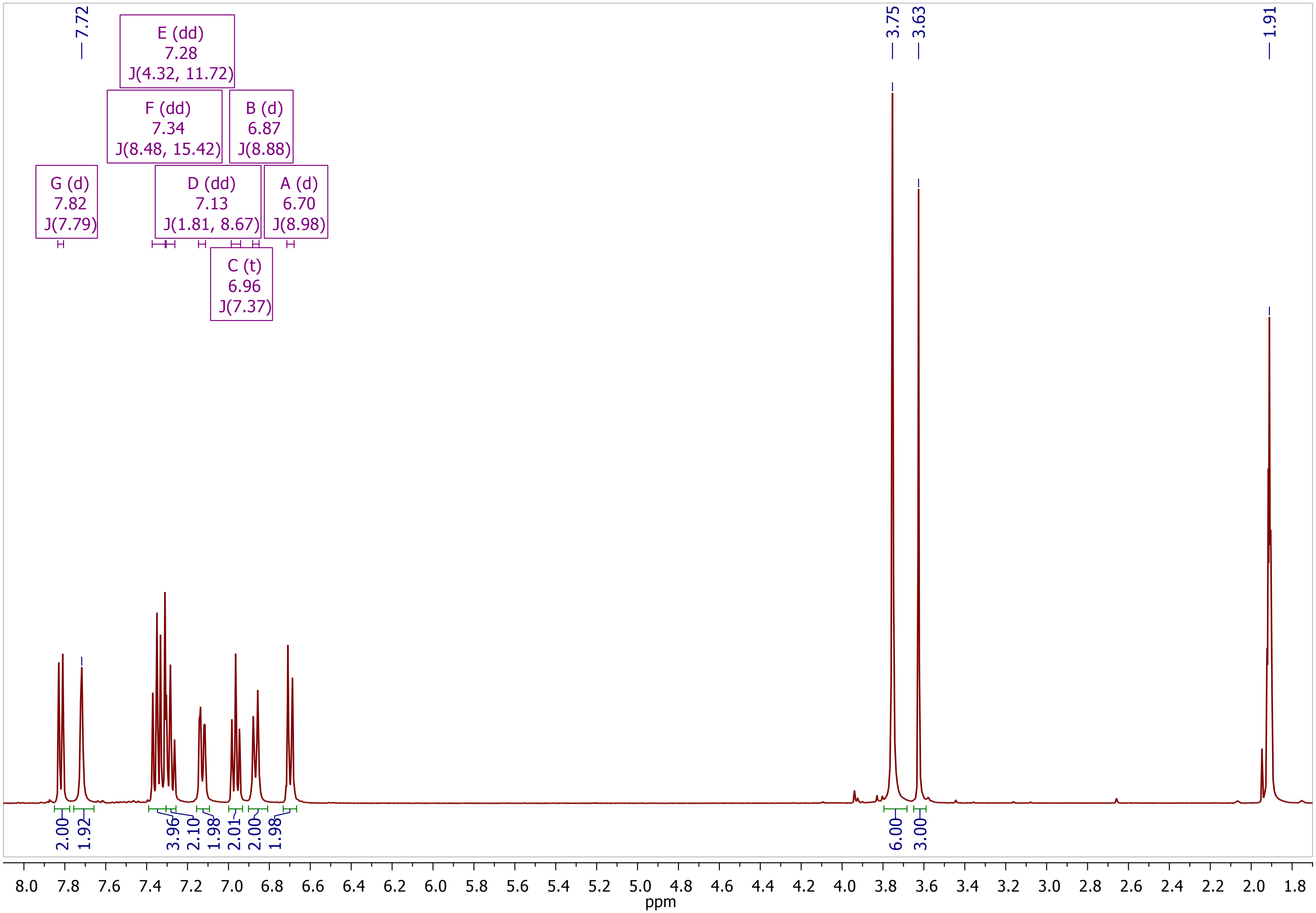

Figure S4. ^{1}H NMR spectrum of **DMCAA** in acetone-d_6.

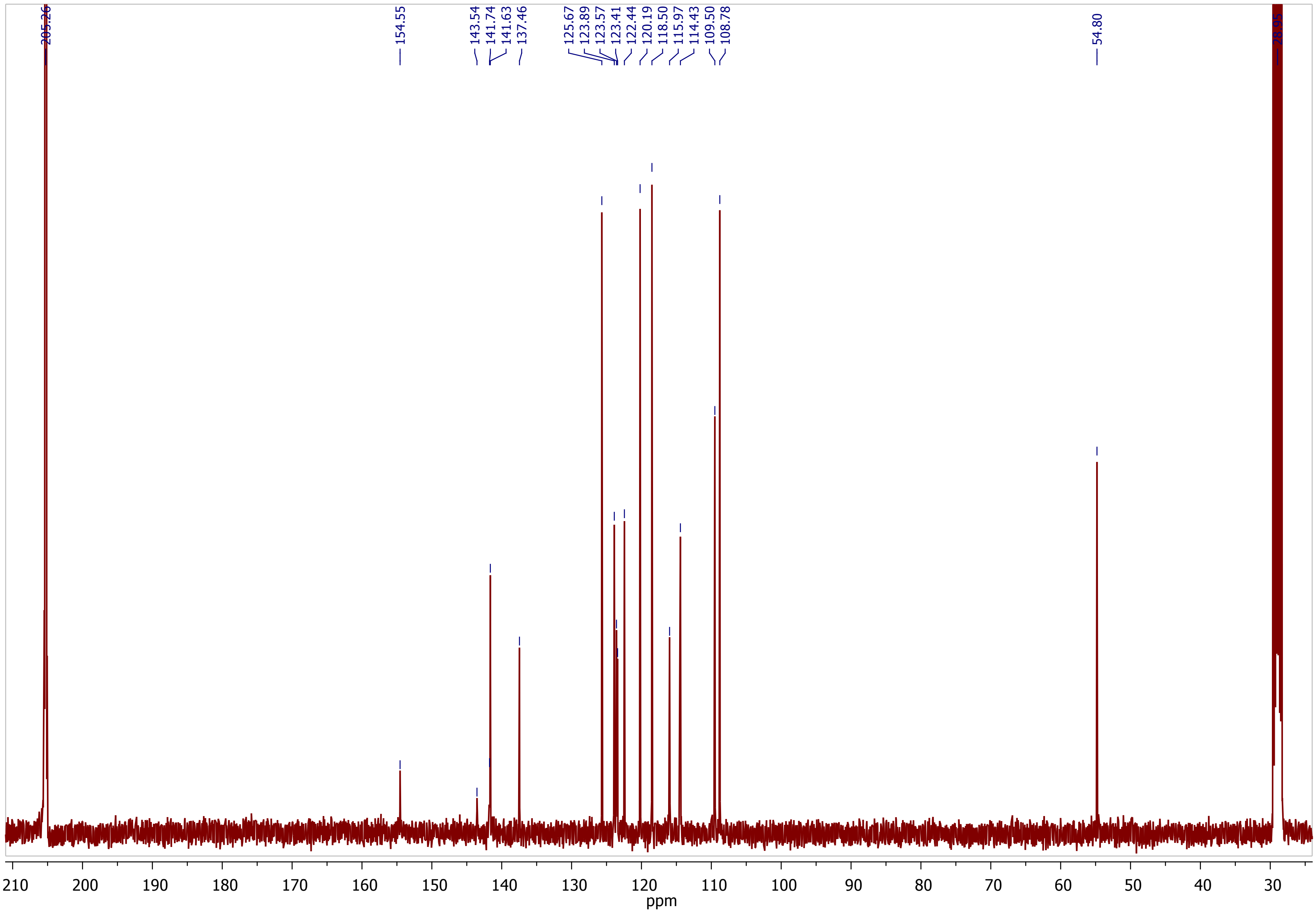

Figure S5. ^{13}C NMR spectrum of **DMCAA** in acetone-d_6.

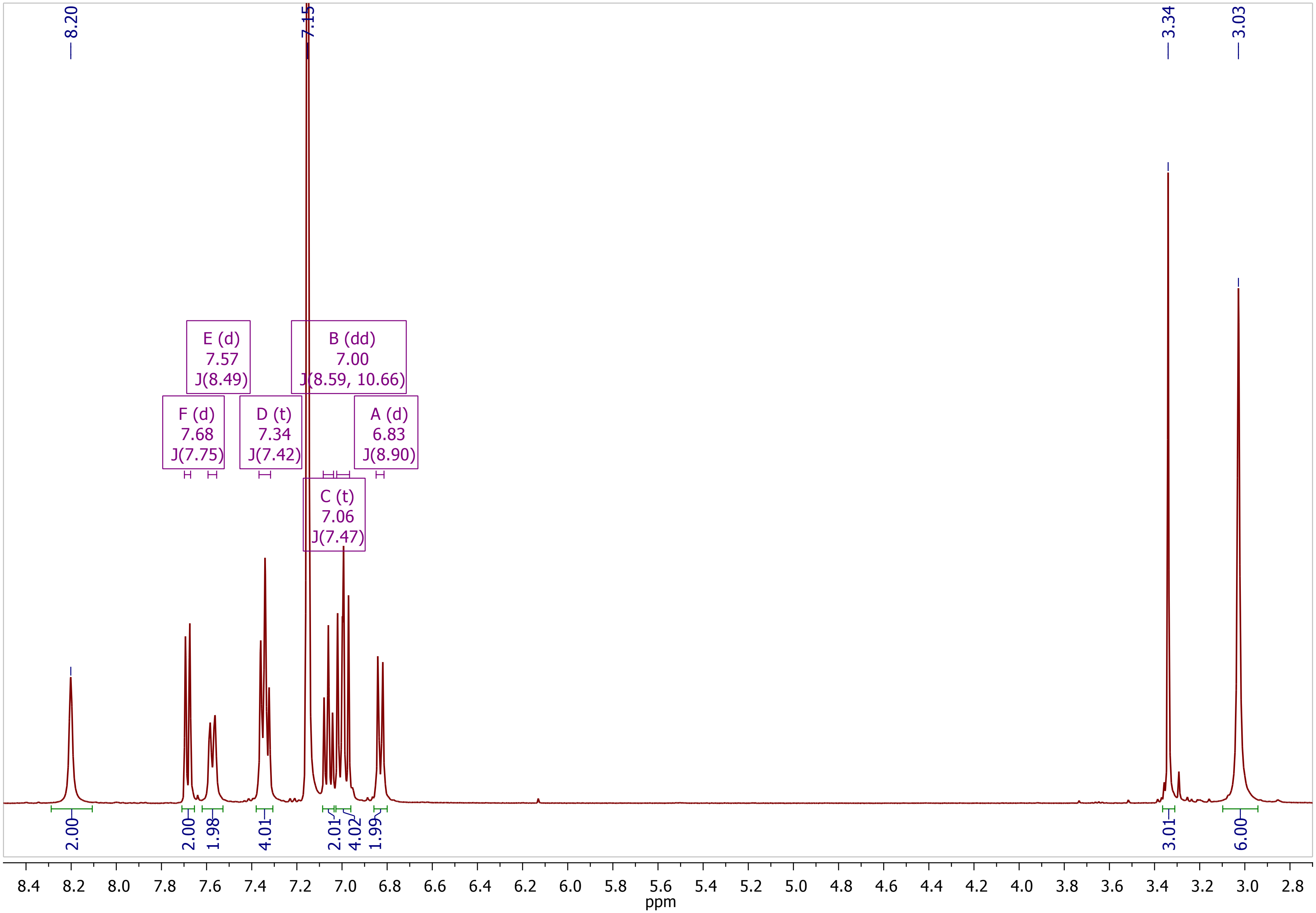

Figure S6. ^{1}H NMR spectrum of **DMCAA** in benzene-d_6.

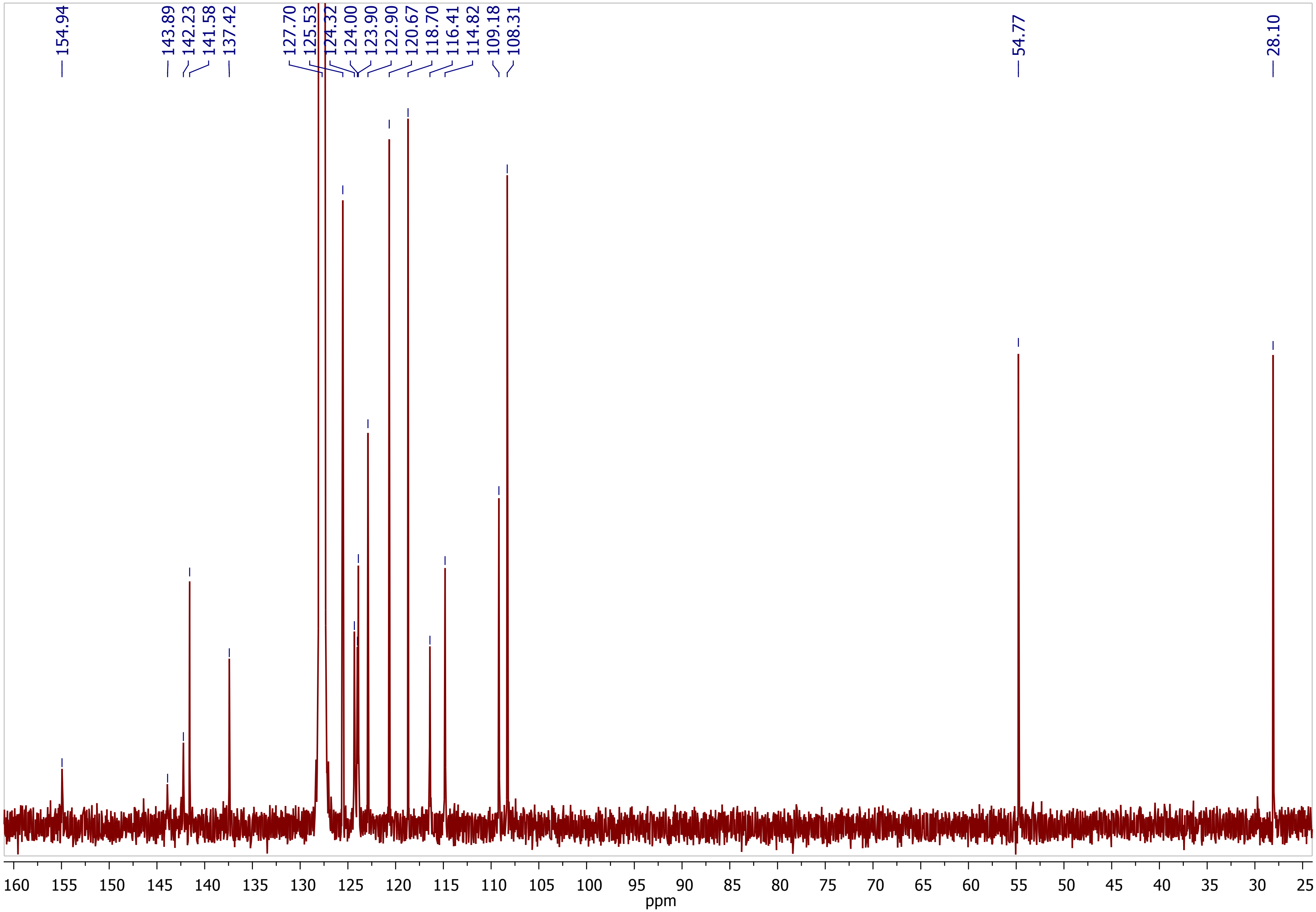

Figure S7. ^{13}C NMR spectrum of **DMCAA** in benzene-d_6.

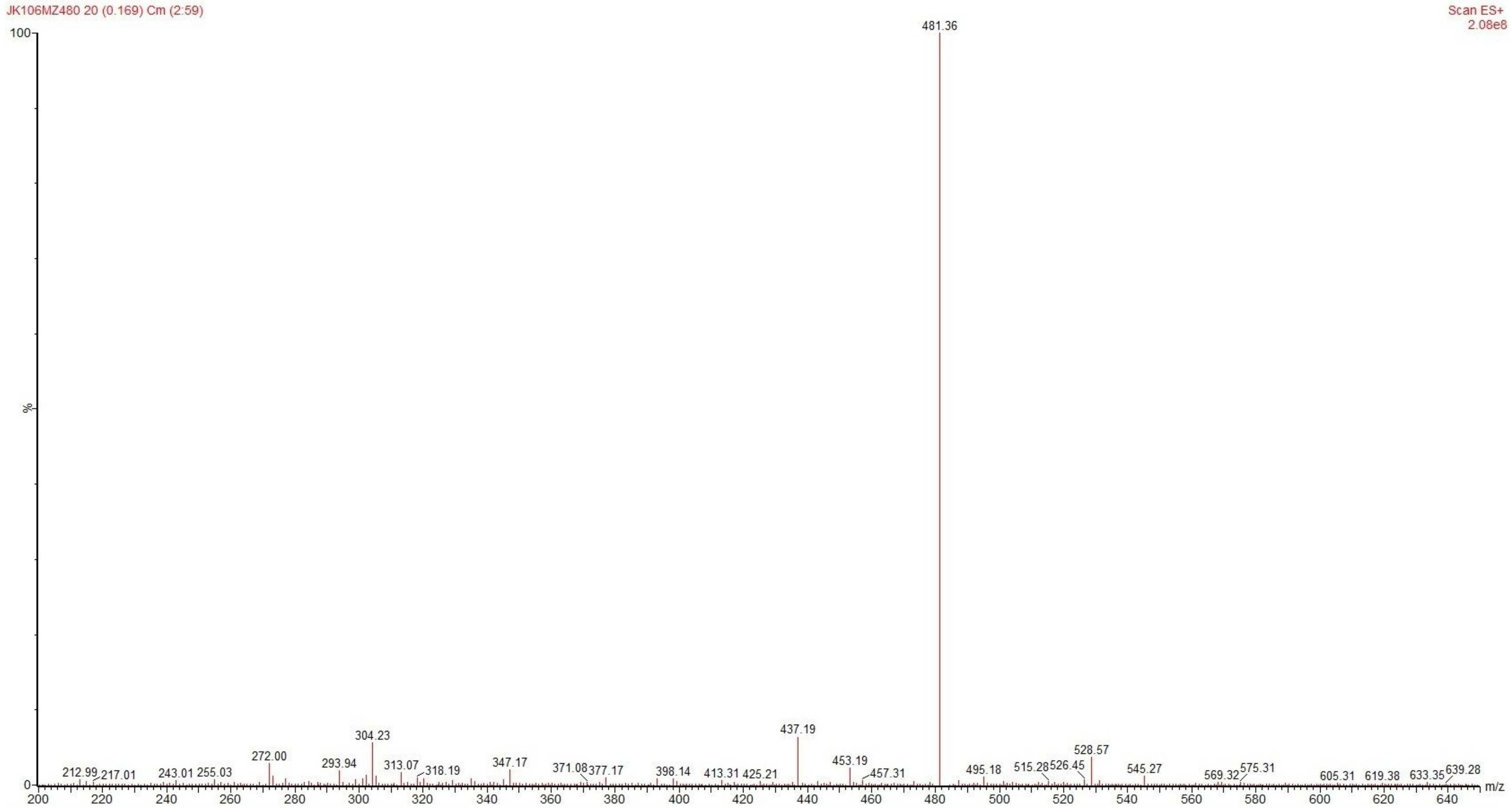

Figure S8. Mass spectrum (MS) of **DMCAA**.

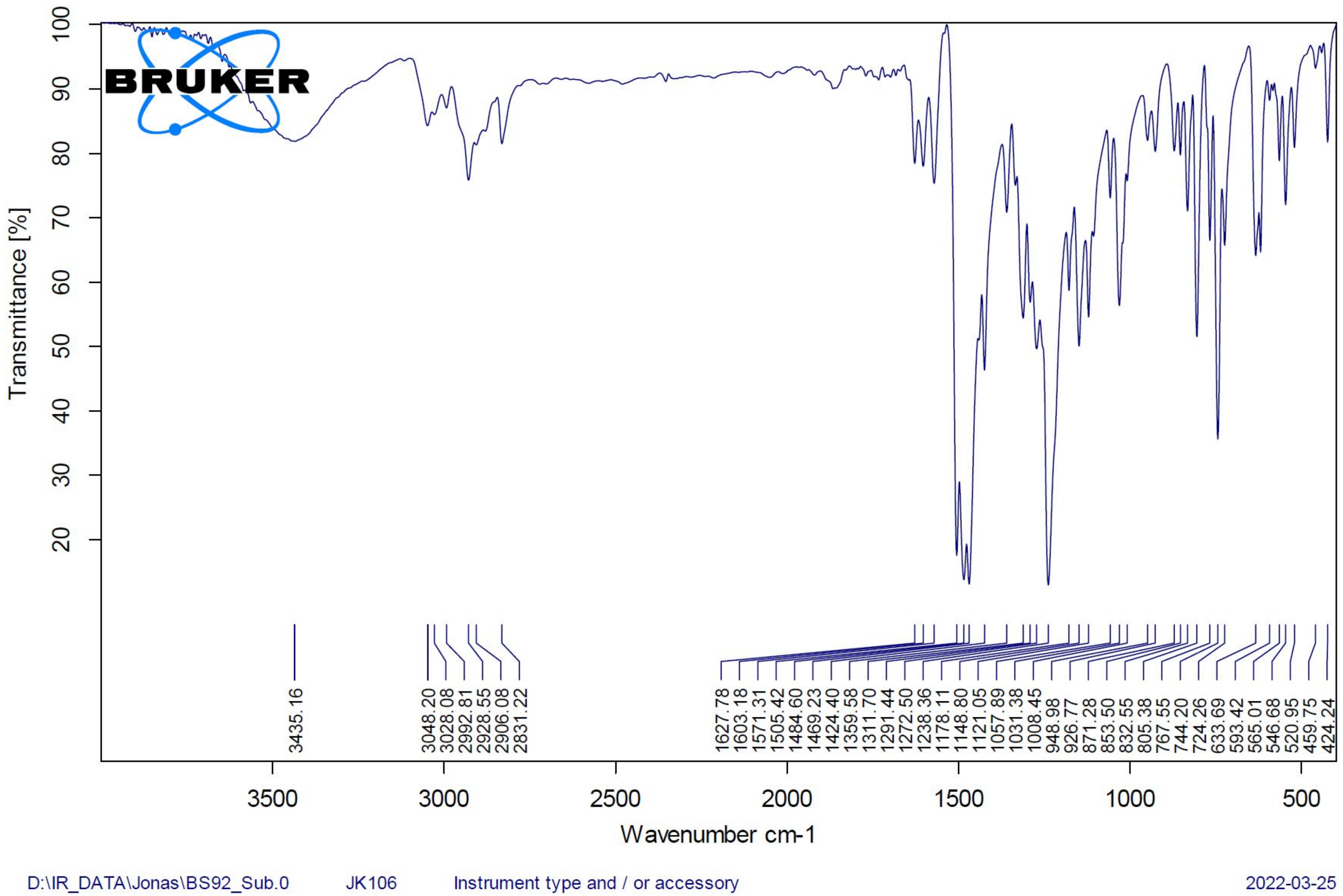

Figure S9. FT-IR spectrum of **DMCAA**.

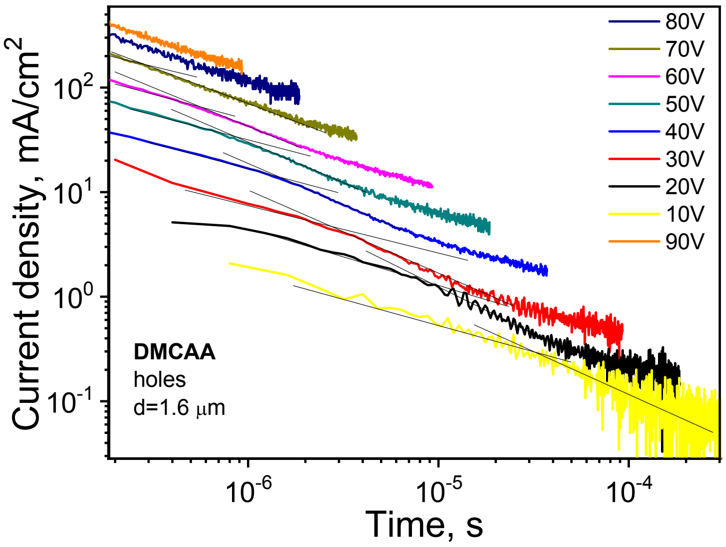

Figure S10. An establishment of transient-times (t_{tr}) for holes (positive current) in double logarithmic scale (both for time and current) from the kink of transient currents.

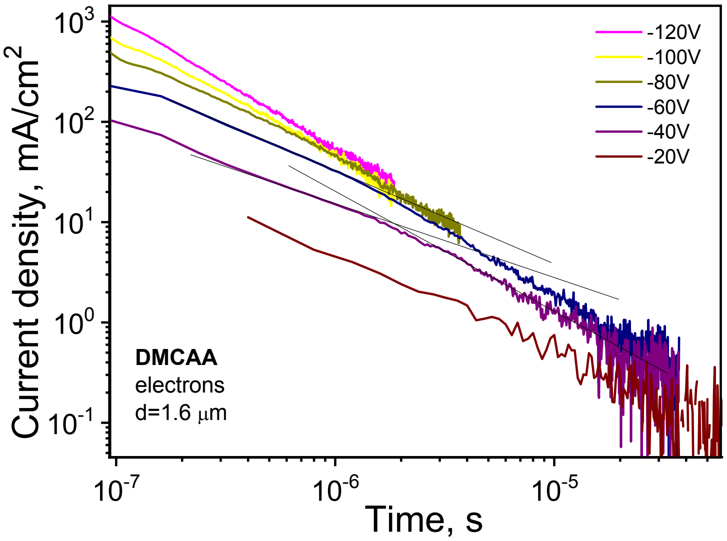

Figure S11. An establishment of transient-times (t_{tr}) for electrons (negative current) in double logarithmic scale (both for time and current) from the kink of transient currents.

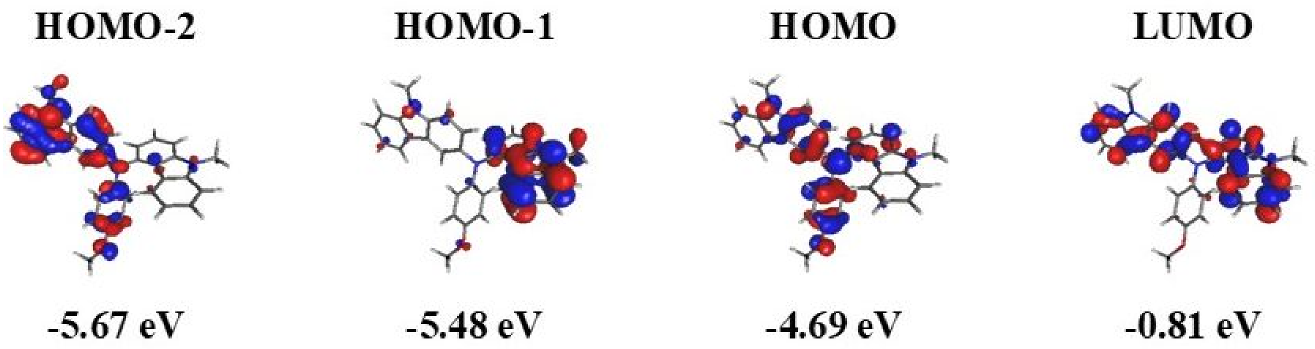

Fig. S12. Shape of frontier orbitals of **DMCAA** calculated at B3LYP/6-31G(d)/CPCM(acetonitrile). The isovalue is equal to 0.03 e^- /au^3 in each case.

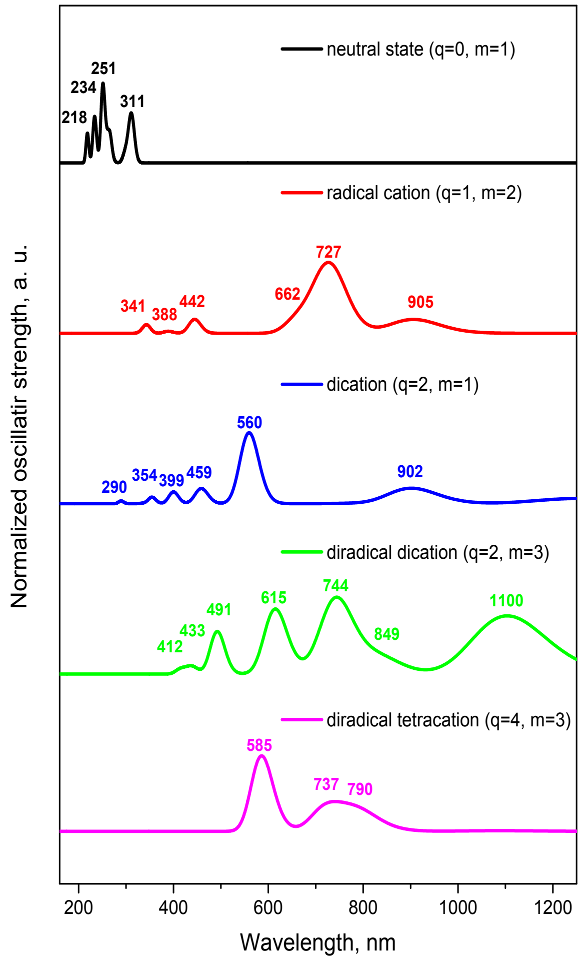

Fig. S13. TDDFT/CAM-B3LYP/6-31G(d)CPCM (acetonitrile) simulated absorption spectra of the different states of **DMCAA.**

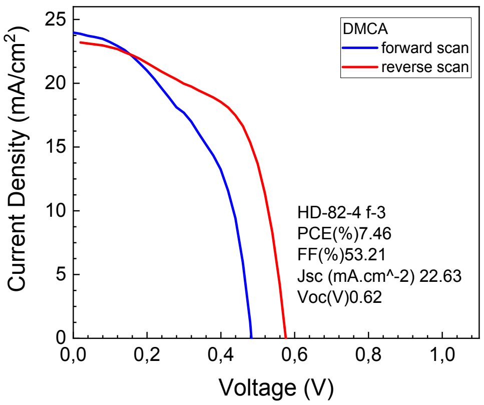

Figure S14. J-V characteristics of p-i-n type devices **DMCAA** without additives. PSCs device measured at forward and reverse scan mode under the simulated sunlight (AM 1.5G, 100 mW cm^2) on the first day. The structure of device is: (Glass/ITO/ DMCAA /FA-MA-Cs (Perovskite)/ PEABr (2D layer)/PCBM/BCP/Au).

References

(1) Yanai, T.; Tew, D. P.; Handy, N. C. A New Hybrid Exchange-Correlation Functional Using the Coulomb-Attenuating Method (CAM-B3LYP). *Chem. Phys. Lett.* **2004**, *393* (1–3), 51–57. https://doi.org/10.1016/J.CPLETT.2004.06.011.

(2) Neese, F. Software Update: The ORCA Program System, Version 4.0. *Wiley Interdisciplinary Reviews: Computational Molecular Science*. John Wiley & Sons, Ltd January 1, 2018, p e1327. https://doi.org/10.1002/WCMS.1327.

(3) Allouche, A. R. Gabedit—A Graphical User Interface for Computational Chemistry Softwares. *Journal of Computational Chemistry*. John Wiley & Sons, Ltd January 15, 2011, pp 174–182. https://doi.org/10.1002/JCC.21600.

(4) Tomasi, J.; Bonaccorsi, R.; Cammi, R.; del Valle, F. J. O. Theoretical Chemistry in Solution. Some Results and Perspectives of the Continuum Methods and in Particular of the Polarizable Continuum Model. *J. Mol. Struct. THEOCHEM* **1991**, *234* (C), 401–424. https://doi.org/10.1016/0166-1280(91)89026-W.

(5) Malinauskas, T.; Tomkute-Luksiene, D.; Sens, R.; Daskeviciene, M.; Send, R.; Wonneberger, H.; Jankauskas, V.; Bruder, I.; Getautis, V. Enhancing Thermal Stability and Lifetime of Solid-State Dye-Sensitized Solar Cells via Molecular Engineering of the Hole-Transporting Material Spiro-OMeTAD. *ACS Appl. Mater. Interfaces* **2015**, *7* (21), 11107–11116. https://doi.org/10.1021/AM5090385/SUPPL_FILE/AM5090385_SI_001.PDF.

(6) Street, R. A. *Hydrogenated Amorphous Silicon*; Cambridge University Press, 1991.

https://doi.org/10.1017/CBO9780511525247.

(7) Tucker, S. H. LXXIV.—Iodination in the Carbazole Series. *J. Chem. Soc.* **1926**, *129* (0), 546–553. https://doi.org/10.1039/JR9262900546.

(8) Grigalevicius, S.; Ma, L.; Qian, G.; Xie, Z.; Forster, M.; Scherf, U. New Carbazole-Based Copolymers as Amorphous Hole-Transporting Materials for Multilayer Light-Emitting Diodes. *Macromol. Chem. Phys.* **2007**, *208* (4), 349–355. https://doi.org/10.1002/MACP.200600498.

(9) Stevens, T. S.; Tucker, S. H. CCXXXVIII.—The Preparation of N-Derivatives in the Carbazole Series. *J. Chem. Soc. Trans.* **1923**, *123* (0), 2140–2147. https://doi.org/10.1039/CT9232302140.

(10) Gauthier, S.; Fréchet, J. M. J. Phase-Transfer Catalysis in the Ullmann Synthesis of Substituted Triphenylamines. *Synth.* **1987**, *1987* (4), 383–385. https://doi.org/10.1055/S-1987-27953/BIB.
